# Oxaliplatin-Induced Senescence in Colorectal Cancer Cells Depends on p14^ARF^-Mediated Sustained p53 Activation

**DOI:** 10.3390/cancers13092019

**Published:** 2021-04-22

**Authors:** Maja T. Tomicic, Franziska Krämer, Alexandra Nguyen, Christian Schwarzenbach, Markus Christmann

**Affiliations:** Department of Toxicology, University Medical Center, Obere Zahlbacher Str. 67, D-55131 Mainz, Germany; franzi.kraemer93@googlemail.com (F.K.); alexandra.nguyen@uni-mainz.de (A.N.); schwarzenbach@uni-mainz.de (C.S.)

**Keywords:** oxaliplatin, cellular senescence, tumor suppressor p53, p14^ARF^, colorectal cancer

## Abstract

**Simple Summary:**

Chemotherapy can lead to cellular senescence in tumor cells. Here we demonstrate that oxaliplatin induces senescence in p53-proficient colorectal cancer (CRC) cells and leads to the G2-phase arrest in all lines studied (HCT116^p53+/+,^ HCT116^p53−/−^, LoVo, SW48, and SW480). At early times the p53-competent lines activate p53 and p21^CIP1^, however, at later times, only LoVo cells showed sustained p53/p21^CIP1^ activation, accompanied by a strong induction of senescence and senescence-associated secretory phenotype (SASP) factors. Opposite to LoVo, the p53/p21^CIP1^ response and senescence induction is much weaker in the other p53-proficient cells, due to p14^ARF^ deficiency. LoVo cells express p14^ARF^ protein and siRNA-mediated knockdown of p14^ARF^ significantly reduces sustained p53/p21^CIP1^ activation and senescence. *Vice versa*, ectopic expression of p14^ARF^ enhances oxaliplatin-induced senescence in SW48 and SW480 cells. Our data show that oxaliplatin-induced senescence in CRC cells depends on p53 proficiency; however, a significant induction can only be observed upon p14^ARF^-mediated p53 stabilization.

**Abstract:**

Senescence is an important consequence of cytostatic drug-based tumor therapy. Here we analyzed to which degree the anticancer drug oxaliplatin induces cell death, cell cycle arrest, and senescence in colorectal cancer (CRC) cells and elucidated the role of p53. Oxaliplatin treatment resulted in the G2-phase arrest in all CRC lines tested (HCT116^p53+/+^, HCT116^p53−/−^, LoVo, SW48 and SW480). Immunoblot analysis showed that within the p53-competent lines p53 and p21^CIP1^ are activated at early times upon oxaliplatin treatment. However, at later times, only LoVo cells showed sustained activation of the p53/p21^CIP1^ pathway, accompanied by a strong induction of senescence as measured by senescence-associated β-Gal staining and induction of senescence-associated secretory phenotype (SASP) factors. Opposite to LoVo, the p53/p21^CIP1^ response and senescence induction was much weaker in the p53-proficient SW48 and SW480 cells, which was due to deficiency for p14^ARF^. Thus, among lines studied only LoVo express p14^ARF^ protein and siRNA-mediated knockdown of p14^ARF^ significantly reduced sustained p53/p21^CIP1^ activation and senescence. *Vice versa*, ectopic p14^ARF^ expression enhanced oxaliplatin-induced senescence in SW48 and SW480 cells. Our data show that oxaliplatin-induced senescence in CRC cells is dependent on p53 proficiency; however, a significant induction can only be observed upon p14^ARF^-mediated p53 stabilization.

## 1. Introduction

The tumor suppressor p53 is, to date, the most important transcription factor, regulating cell cycle arrest, cell survival, and cell death. A specific form of cell cycle arrest is cellular senescence, which was initially described as a permanent cell cycle arrest that limits the life span of cultured human fibroblasts [1]. Contrary to quiescence, which is defined as a temporary cell cycle arrest, senescence is not reversible in response to proliferative conditions (for review see [2,3,4,5]). Cellular senescence can be induced by telomere shortening (replicative senescence), genomic damage, proliferation-associated signals (oncogenic senescence), and epigenetic alterations, and comprises pleiotropic effects, not only leading to tumor suppressive mechanisms, but also to age-associated degenerative pathologies via promoting chronic inflammation. In all cases, activation of senescence depends on persisting DNA damage and chronic DNA damage response (DDR) activation. DNA damage-induced senescence is directly activated by various insults; however, the exact kind of DNA damage that induces senescence is still unclear. Most likely, all kinds of damages have to be converted into unrepairable double-strand breaks (DSBs). During replicative senescence, telomeres are constantly shortened and can be recognized as DSBs, whereas during oncogenic senescence, mutations in oncogenes lead to the activation of positive cell cycle regulators, driving cells into excessive *ori* firing and re-replication, and finally, via breakdown of the replication forks to the formation of DSBs. In all circumstances, the unrepaired DSBs trigger a chronic activation of the DDR and via ATR/ATM signaling induction of p53 and CDKN1A/p21^CIP1^, eventually inducing senescence (for review see [6,7]). Transcriptional activation of p21^CIP1^ is the main cause of senescence induction. p21^CIP1^ arrests the cell cycle via inhibition of various cyclin-dependent kinases (CDKs) [8,9]. Besides p21^CIP1^, two additional factors are important for senescence induction and maintenance, namely p14^ARF^ and p16^INK4A^ [10,11]. Both factors are transcribed from the *CDKN2A* gene. Whereas p16^INK4a^ inhibits CDK4 and CDK6 [12], p14^ARF^ acts as a stabilizer for p53, as it can sequester MDM2, a protein responsible for the degradation of p53 [13,14,15].

An important type of DNA damage-induced senescence is related to anticancer therapy [16]. In the case of anticancer drugs, senescence is not dependent on telomere shortening, but rather on oxidative stress and/or unrepaired DNA damage and is also referred to as stress-induced premature cellular senescence (SIPS), accelerated cellular senescence (ACS), or senescence-like phenotype [17,18]. For anticancer drug-induced senescence, less data is available concerning the underlying mechanisms, as compared to replicative and oncogenic senescence. Previously, we showed that, upon exposure to the anticancer drug temozolomide, glioblastoma cells predominantly entered senescence instead of undergoing cell death [19]. Temozolomide-induced senescence, but not cell cycle arrest, required functional p53, and was dependent on sustained p21^CIP1^ induction. Here, we address the question whether the dominant role of p53/p21^CIP1^ activation for DNA-damage-induced senescence can also be observed upon treatment with oxaliplatin in colorectal carcinoma (CRC) cell lines. Oxaliplatin binds predominantly the N7 nitrogen of the purine bases guanine and adenine leading to mono- and bifunctional adducts, which are converted into intra-strand cross-links (Pt-GG adducts, Pt-AG adducts, and G-Pt-G adducts), as well as inter-strand crosslinks leading to cell cycle arrest and cell death [20,21,22,23]. Whereas the cytotoxic activity of oxaliplatin has been analyzed in multiple studies, not much is known concerning the impact of senescence in the oxaliplatin response of CRC cells.

Therefore, we analyzed whether (and to which extent) oxaliplatin induces senescence in CRC cell lines and analyzed the role of p53, p21^CIP1^, p14^ARF^, and p16^INK4a^ in this process. Our results indicate that only a fraction of CRC cells enters senescence upon oxaliplatin and that senescence depends on p53/p21^CIP1^ and p14^ARF^ proficiency.

## 2. Materials and Methods

### 2.1. Cell Culture, Drug Treatment, siRNA-Mediated Knockdown, and Plasmid Transfection

CRC cell lines (LoVo, SW48, SW480) were ATCC-purchased and cultured in RPMI medium supplemented with 10% fetal bovine serum (FBS, Gibco, Thermo Fisher Scientific, Waltham, MA, USA) at 37 °C, 6% CO_2_. HCT116^p53+/+^, and HCT116^p53−/−^ cells were kindly provided by Professor Bert Vogelstein (Johns Hopkins University, Baltimore, ML, USA). The cell lines were kept in culture for max. 2 month and were regularly checked for mycoplasma contamination using the VenorGEM classic detection kit (#11-1100) from Minerva Biologicals. Oxaliplatin and irinotecan (CPT-11) were prepared as 5 mg/mL and 20 mg/mL stock solution, respectively, by the pharmacy of the University Medical Center Mainz. Pifithrin α (PTHα, P4359, Sigma-Aldrich, St. Louis, MO, USA) was used at 30 µM. For silencing CDKN2a, predesigned siRNA (s217, Thermo Fisher Scientific) and control human non-silencing siRNA (Silencer Select Predesigned siRNA Negative Control #1 siRNA; Ambion, Austin, TX, USA) were used. For re-expressing p14^ARF^, SW48 and SW480 cells were transfected with the pcDNA3-myc-ARF plasmid (19930, Addgene, [14]) or, as control, pcDNA3 using Effectene Reagent (Qiagen, Hilden, Germany).

### 2.2. Determination of Cell Death, Cell Cycle Progression, and Colony Formation Capacity

To determine cell death and cell cycle distribution, cells were incubated for different times after drug exposure, stained with propidium iodide (PI) and subjected to flow cytometry, as described [24]. The data were analyzed using the CellQuestPro/FACS DIVA Software (BD Canto II). For colony formation assays, 1 × 10^3^ cells were seeded per 6-cm dish and 24 h later exposed to anticancer drugs, as described [25]. Colonies were fixed in methanol and stained with 1.25% Giemsa/0.125% crystal violet three weeks after exposure.

### 2.3. Determination of Senescence

For detection of senescence via β-Gal staining, cells were washed twice with PBS, afterwards fixed with 2% formaldehyde, 0.2% glutaraldehyde in PBS. After washing with PBS, cells were stained (40 mM citric acid/phosphate buffer pH 6.0, 150 mM NaCl, 2 mM MgCl_2_, 5 mM potassium ferrocyanide, 5 mM potassium ferricyanide, 0.1% x-Gal) overnight at 37 °C. Cells were then washed with PBS and overlaid with 70% glycerine. Micrographs were acquired and analyzed using the ECHO Rebel microscope (Discover ECHO INC., San Diego, CA, USA). For detection of senescence via C_12_FDG staining, cells were exposed to oxaliplatin for indicated times. Thereafter, medium was removed and 4 mL fresh medium containing 10% FBS and 300 µM chloroquine was added per 10 cm dish. Cells were incubated for 30 min at 37 °C. Finally, 33 µM C_12_FDG (ImaGene Green™ C_12_FDG lacZ Gene Expression Kit) was added and incubated for 90 min at 37 °C. The medium was removed; cells were washed with PBS, trypsinized, and resuspended in PBS. Senescence was measured at the BD FACSCanto™ II flow cytometer. 

### 2.4. Preparation of RNA and RT-qPCR 

Total RNA was isolated using the Nucleo Spin RNA Kit (Machery and Nagel, Düren, Germany). One µg total RNA was transcribed into cDNA (Verso cDNA Kit, Thermo Scientific) and qPCR was performed using GoTaq^®^ qPCR Master Mix (Promega, Madison, WI, USA) and the CFX96 Real-Time PCR Detection System (Bio-Rad, Munich, Germany). The analysis was performed using CFX Manager^TM^ software. Non-transcribed controls were included in each run, expression was normalized to *GAPDH* and *ACTB*; the untreated control was set to one. The specific primers are listed in Table A1.

### 2.5. Preparation of Genomic DNA and Methylation-Specific PCR (MSP) 

DNA was extracted according to standard protocols using phenol-chloroform extraction. Modification of the DNA was performed using the EZ DNA Methylation Kit from Zymo Research. Methylation specific PCR was performed using following primer sequences (5′-3′): p14^ARF^: Meth-up GTGTTAAAG GGCGGCGTAGC, Meth-low AAAACCCTCACTCGCGACGA, Unmeth-up TTTTTGGTGTTAAAGGGTGGTGTAGT, Unmeth-low CACAAAAACCCTCACTCACAACAA; p16^INK4A^: Meth-up TTATTAGAGGGTGGGGCGGATCGC, Meth-low GACCCCGAACCGCGACCGTAA, Unmeth-up TTATTAGAGGGTGGGGTGGATTGT Unmeth-low CAACCCCAAACCACAACCATAA; p14^ARF^ [26] and p16^INK4A^ [27] specific primers have been published before. As positive control for the reaction, the β-actin promoter was included. Actin-up: AGGGAGTATATAGGTTGGGGAAGTT, Actin-low AACACACAATAACAAACACAAATTCAC. 

### 2.6. Preparation of Cell Extracts and Western-Blot Analysis

Whole-cell extracts were prepared by direct lysis with 1x loading buffer (Roti-Load^®^, Roth) heated to 95 °C. Proteins were separated by SDS-PAGE and transferred onto a nitrocellulose membrane (Amersham) by western blotting, blocked in 5% BSA dissolved in TBS-Tween and incubated with specific primary antibodies and secondary peroxidase conjugated antibodies (Table A2). Detection was performed using the iBright CL1000 (Invitrogen) system.

### 2.7. Statistical Analysis 

The data were evaluated using Student’s t-test and were expressed as a mean ± SD * *p* ≤ 0.1 significant, ** *p* ≤ 0.01 very significant, *** *p* ≤ 0.001 highly significant. Statistical analyses were performed using GraphPad Prism version 6.01 for Windows, GraphPad Software, La Jolla, CA, USA. 

## 3. Results

### 3.1. Oxaliplatin Induces Cell Death and Cell Cycle Arrest

**Figure 1:**
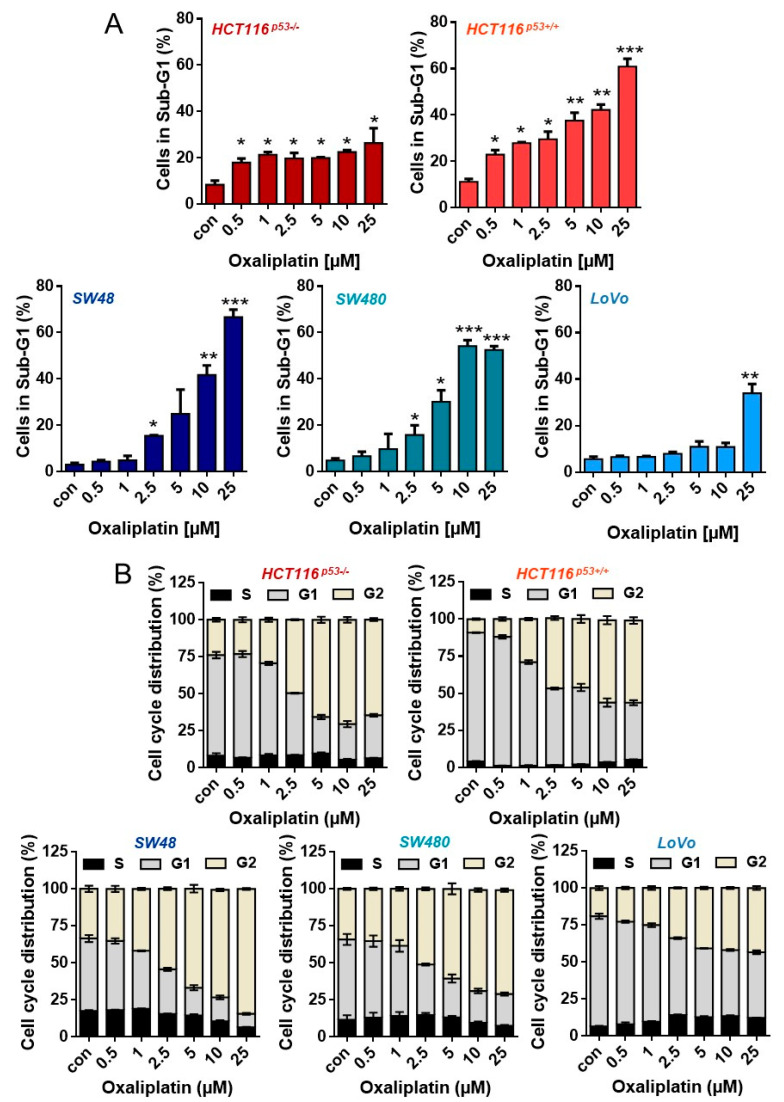
(**A**) Cell death and (**B**) cell cycle distribution was measured by flow cytometry 96 h upon exposure to different concentrations of oxaliplatin in PI-stained CRC cells. Experiments were performed in triplicates and differences between treatment and control were statistically analyzed using Student’s t test (not labeled = not significant, * *p* < 0.1 ** *p* < 0.01, *** *p* < 0.001). Concerning cell cycle distribution, at concentrations > 2.5 µM, a significant (* or **) decrease of cells in G1 and increase in the G2-phase was observed.

**Figure A1:**
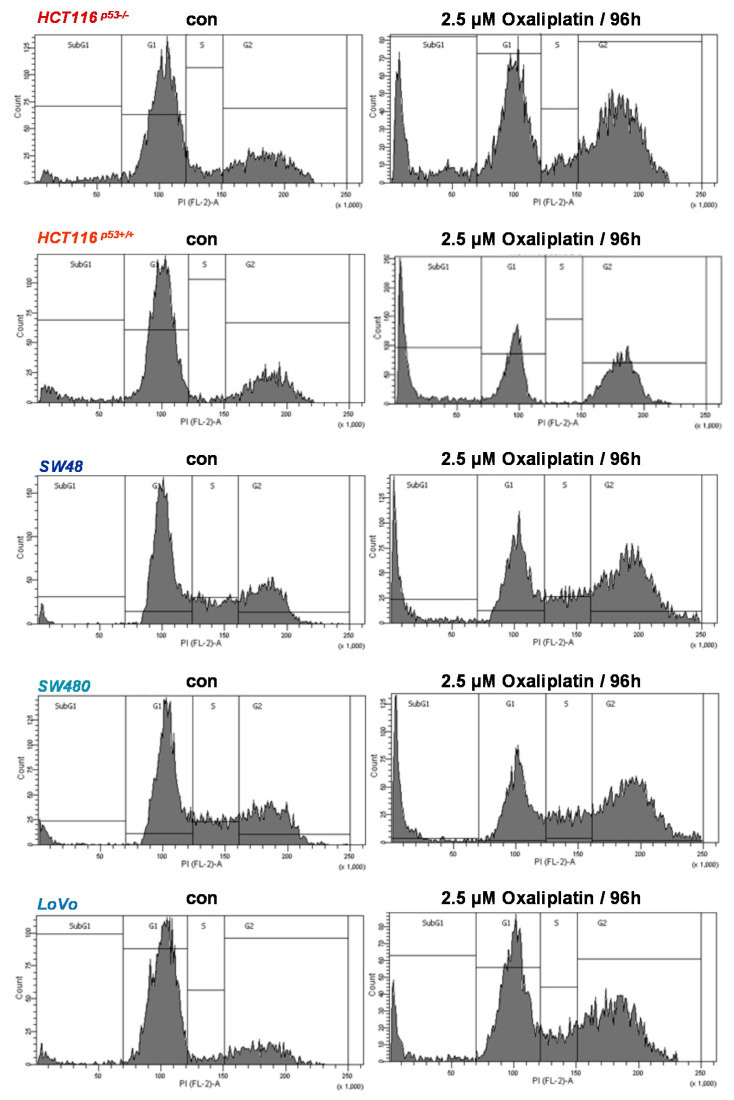
Cell death and cell cycle distribution were measured by flow cytometry 96 h upon exposure to oxaliplatin in PI-stained CRC cells. Representative histograms were shown for untreated cells (con) and cells exposed to 2.5 µM oxaliplatin.

To analyze whether (and to which degree) oxaliplatin induces senescence in CRC cells, we utilized five different cell lines differing in their p53 status. Thus, HCT116^p53+/+^, LoVo and SW48 are p53-proficient (wild type); SW480 harbors a p53-stabilizing mutation, but retains p53-dependent transcriptional activity. Opposite, HCT116^p53−/−^ cells were deficient for p53. First, we analyzed sensitivity of these cell lines to oxaliplatin to identify moderate toxic concentrations. The results show that among the cell lines, LoVo were most resistant, showing no toxicity up to 10 µM oxaliplatin, 96 h after exposure. After treatment with 25 µM oxaliplatin, LoVo cells showed ~40% dead cells (Figure 1A). The same level of toxicity was achieved in HCT116^p53+/+^ and SW480 cells upon exposure to 5 µM, as well as in SW48 cells after treatment with 10 µM oxaliplatin. In SW48 and SW480 cells, the first signs of toxicity were observed after exposure to 2.5 µM oxaliplatin. Opposite, HCT116^p53+/+^ already showed toxicity after 0.5 µM oxaliplatin. Direct comparison between the isogenic p53-proficient and p53-deficient HCT116 cell lines showed that particularly HCT116^p53+/+^ cells were highly sensitive to oxaliplatin. Similar to HCT116^p53+/+^ cells, HCT116^p53−/−^ cells showed toxicity after 0.5 µM oxaliplatin, however, the toxicity did not significantly rise upon treatment with higher oxaliplatin concentrations. Independent of the cell line, an accumulation of the cells in the G2-phase was observed (Figure 1B and Figure A1 for histograms). The weakest accumulation in G2 was observed in LoVo cells.

### 3.2. Oxaliplatin Induces Proliferation Arrest and Senescence

**Figure 2:**
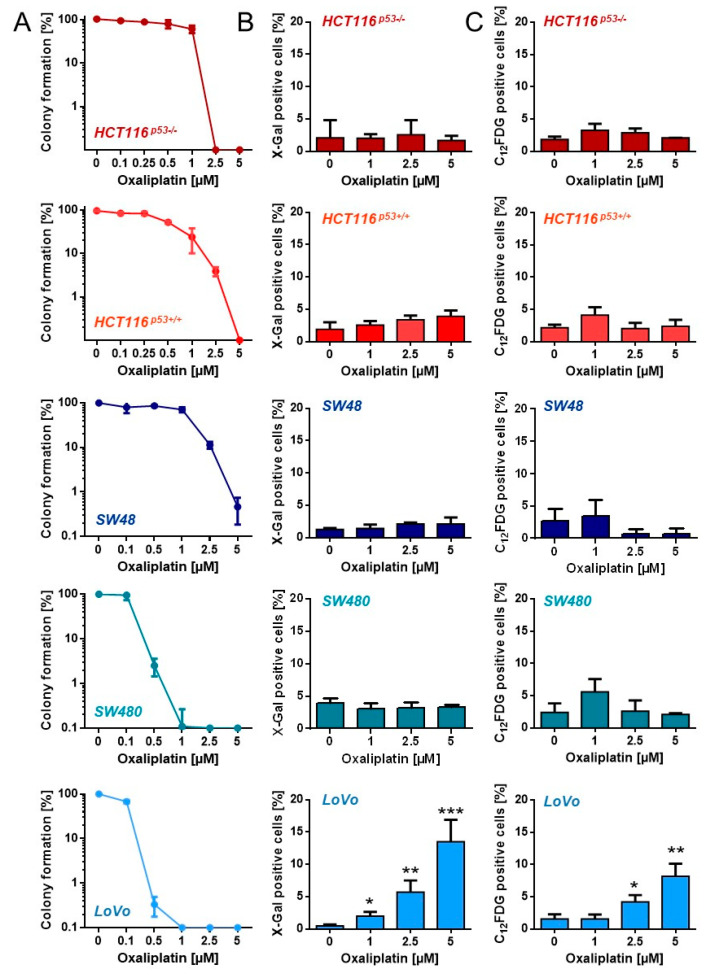
(**A**) Proliferation arrest was measured upon exposure to different oxaliplatin concentrations by CFA in CRC cells. (**B**) Senescence was measured microscopically by detection of β-Gal positive cells 120 h after oxaliplatin exposure. (**C**) Senescence was measured by flow cytometry using C_12_FDG staining 120 h after oxaliplatin exposure. (**B**,**C**) Experiments were performed in triplicates and differences between treatment and control were statistically analyzed using Student’s t test (not labeled = not significant, * *p* < 0.1, ** *p* < 0.01, *** *p* < 0.001).

To analyze at which concentrations oxaliplatin abrogates cellular proliferation, we performed colony formation assays (Figure 2A). A significant loss of the proliferation ability (≤10%) was observed in HCT116^p53+/+^, HCT116^p53−/−^ and SW48 cells after treatment with 2.5 µM oxaliplatin. SW480 and LoVo cells already showed a dramatic loss in colony formation after exposure to 0.5 µM oxaliplatin. Next, senescence induction was analyzed using microscopy-based detection of β-Gal positive cells (Figure 2B) and FACS-based detection of C_12_FDG positive cells (Figure 2C). Among all cell lines, a significant induction of senescence was only observed in LoVo cells. However, the fraction of senescent cells was rather low, reaching only 10–15%.

### 3.3. Oxaliplatin Induces Cell Death, Cell Cycle Arrest, and Senescence at Late Time Points

**Figure 3:**
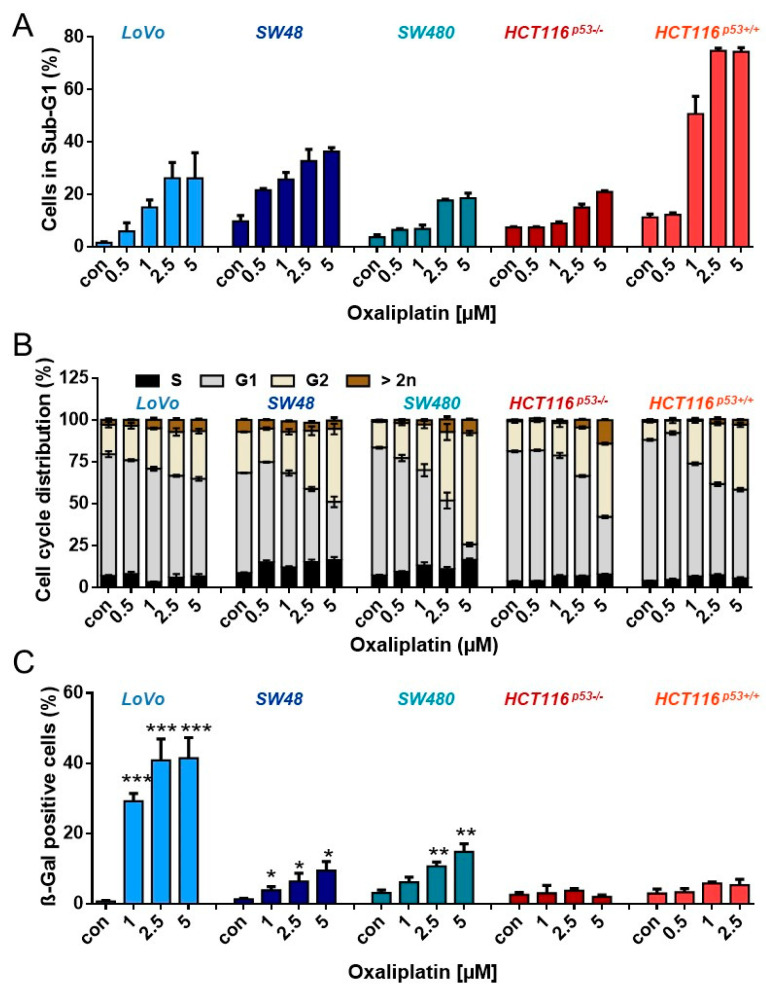
(**A**) Cell death and (**B**) cell cycle distribution was measured by flow cytometry 168 h upon exposure to different concentrations of oxaliplatin in PI-stained CRC cells. (**C**) Senescence was measured microscopically by detection of β-Gal positive cells 168 h after oxaliplatin exposure. (**A**–**C**) Experiments were performed in triplicates and differences between treatment and control were statistically analyzed using Student’s t test (not labeled = not significant, * *p* < 0.1, ** *p*< 0.01, *** *p* < 0.001). Concerning cell cycle distribution, at concentrations > 2.5 µM, a significant (* or **) decrease of cells in G1 and increase in the G2-phase was observed.

Since the low frequency of senescent cells could be explained by oxaliplatin-induced senescence being a very late response, we analyzed cell death, cell cycle arrest, and senescence one week (168 h) after treatment. As expected, the observed toxicity was higher than after 96 h. Thus, ~ 20–30% of LoVo, SW48, SW480 and HCT116^p53-/-^ cells died after treatment with 2.5 µM oxaliplatin (Figure 3A). Again, HCT116^p53+/+^ were most sensitive, showing ~75% dead cells after 2.5 µM oxaliplatin. In analogy to shorter exposure to oxaliplatin, an accumulation of the cells in the G2-phase was also observed after longer exposure (Figure 3B). Similar to the early time points, LoVo cells showed a significant induction of senescence upon treatment with 2.5 and 5 µM oxaliplatin, reaching ~40% (Figure 3C). Those oxaliplatin concentrations also induced senescence in SW48 and SW480 cells; however, the frequency was much lower, with 10% in SW48 and 15% in SW480 cells.

### 3.4. Oxaliplatin Induces Upregulation of SASP and SCAP Factors

**Figure 4:**
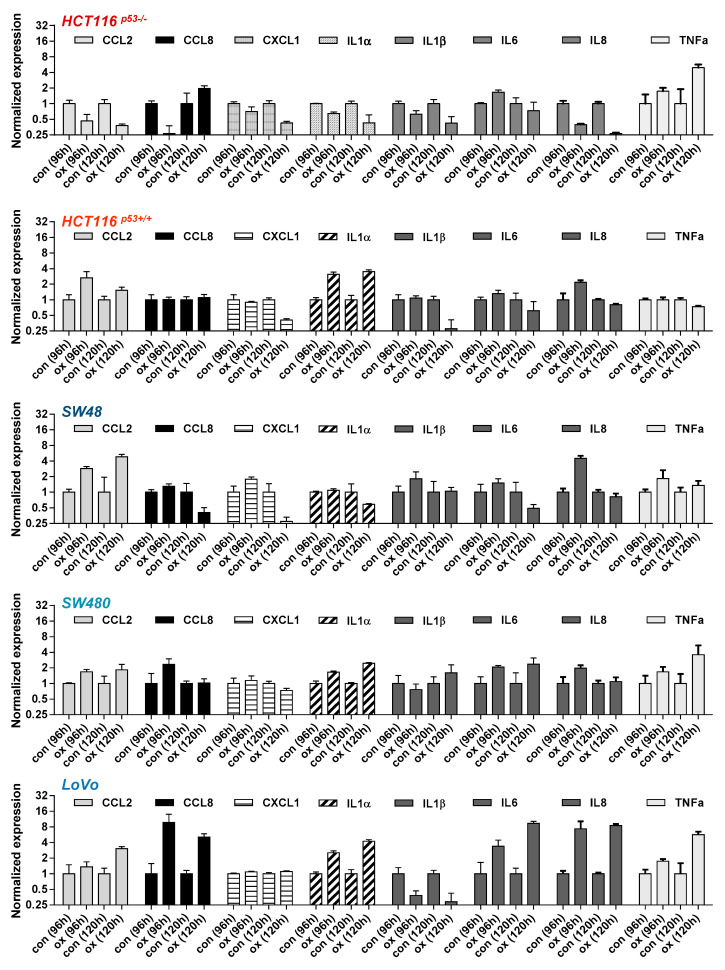
CRC cells were treated with 2.5 µM oxaliplatin. 96 and 120 h later, expression of the SASP factors *CCL2*, *CCL8*, *CXCL1*, *IL1α*, *IL1β*, *IL6*, *IL8*, and *TNFα* mRNA was measured by qPCR.

To verify the results obtained by β-Gal and C_12_FDG staining, we next measured the activation of the senescence-associated secretory phenotype (SASP), which is characterized by the induction and secretion of different cytokines [28] and represents a clinically relevant mechanism affecting the outcome of cancer therapy. In order to analyze whether oxaliplatin can induce the SASP, the time-dependent expression of different SASP components was analyzed (Figure 4). 

In HCT116^p53+/+^, SW48 and SW48 cells a weak induction of *IL8* and *CCL2* (2-4-fold) was observed after exposure to 2.5 µM oxaliplatin. A weak induction of *CCL2* in LoVo cells was observed at 120 h (3-fold), whereas a strong induction of *IL8* (8-fold) was observed both at 96 and 120 h. Furthermore, LoVo cells also exhibited an upregulation of *CCL8* and *IL6* (4-8 fold); induction of *IL1α* was observed in HCT116^p53+/+^, SW480 and LoVo cells. Overall, similar to the results on senescence induction obtained by β-Gal and C_12_FDG staining, the strongest activation of the SASP factors was observed in LoVo cells.

**Figure A2:**
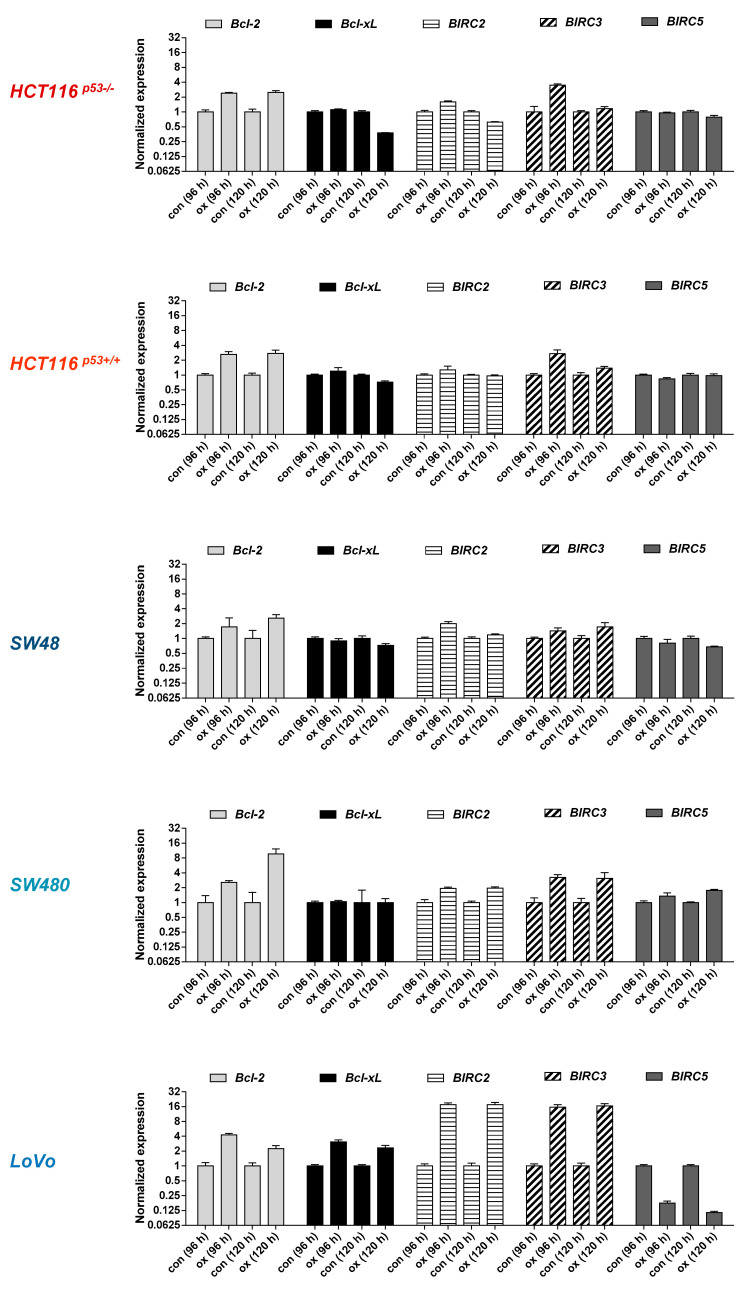
CRC cells were treated with 2.5 µM oxaliplatin. 96 and 120 h later, expression of the anti-apoptotic factors *Bcl-2*, *Bcl-xL*, *BIRC2 (c-IAP1)*, *BIRC3 (c-IAP2)*, and *BIRC5 (Survivin)* was measured by qPCR.

**Figure A3:**
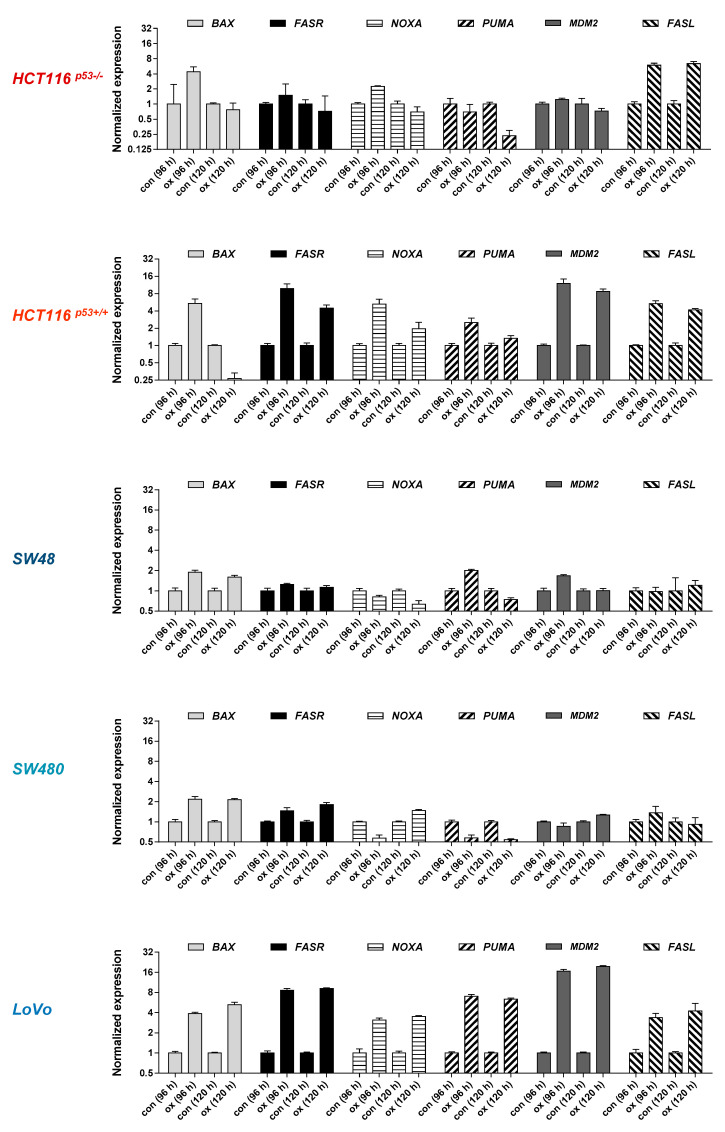
CRC cells were treated with 2.5 µM oxaliplatin. 96 and 120 h later, expression of the pro-apoptotic factors *BAX*, *FASR*, *NOXA*, *PUMA*, *FASL*, and of the p53 target *MDM2* was measured by qPCR.

An important mechanism leading to senescence induction and its maintenance is the upregulation of anti-apoptotic factors, rendering senescent cells resistant to apoptosis. This mechanism is referred to as senescent-cell anti-apoptotic pathway (SCAP) which might be caused by senescence-associated mitochondrial dysfunction (SAMD) (for review see [29,30]). In order to analyze whether oxaliplatin induces the SCAP factors, the time-dependent expression of different anti-apoptotic factors was analyzed by real-time qPCR (Figure A2). In all cell lines, a weak (2-fold) transcriptional activation of *BcL-2* was observed at 120 h. SW480 cells exhibited an 8-fold *Bcl-2* induction. Of note, a strong difference in the expression of *BIRC2* (encodes c-IAP1), *BIRC3* (encodes c-IAP2) and *BIRC5* (encodes Survivin) was observed in LoVo cells, with a 16-fold upregulation of *BIRC2* and *BIRC3* and an 8-fold downregulation of *BIRC5*. This pronounced transcriptional repression of the *Survivin* gene also shows that the cells must be significantly compromised in their proliferation potential, presumably undergoing senescence [31,32]. Beside anti-apoptotic factors, we also compared the transcriptional activation of pro-apoptotic factors among the CRC cell lines upon oxaliplatin (Figure A3). Also, in this case, clear differences were observed. Corresponding to the high toxicity, an induction of the genes coding for the p53-dependent pro-apoptotic factors *FASR*, *PUMA*, and *NOXA*, as well as of the p53 target *MDM2* and the AP1-dependent pro-apoptotic factor *FASL* was observed in HCT116^p53+/+^ cells, whereas HCT116^p53−/−^ cells only showed induction of the *FASL* mRNA. Interestingly the highest induction of all these factors, and of the p53-dependent pro-apoptotic factor BAX, was observed in LoVo cells, indicating a more efficient activation of the p53 pathway than in SW48 and SW480 cells.

### 3.5. Activation of p53 and p21^CIP1^ upon Oxaliplatin Treatment

**Figure 5:**
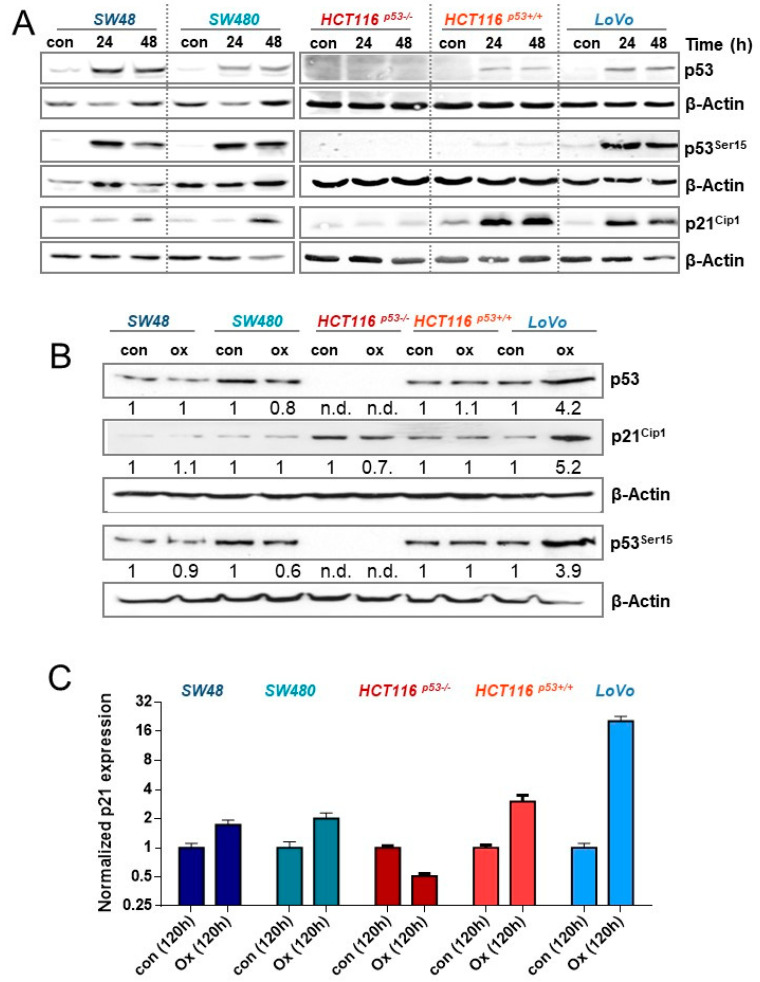
(**A**) CRC cells were treated with 2.5 µM oxaliplatin; 24 and 48 h later, expression of p53 and p21^CIP1^, as well as the phosphorylation of p53 at Ser15, was measured by immunodetection. (**B**) CRC cells were treated with 2.5 µM oxaliplatin. 120 h later, the expression of p53 and p21^CIP1^, as well as the phosphorylation of p53 at Ser15 was measured by immunodetection. (**C**) CRC cells were treated with 2.5 µM oxaliplatin. 120 h later, the expression of *p21^CIP1^* mRNA was determined by qPCR. (**A**,**B**) β-Actin was used as internal loading control, (**B**) x-fold induction was measured densitometrically and is annotated under the respective blot.

One possibility to explain the different efficiency of oxaliplatin to induce senescence in CRC cells would be a dissimilar activation of the DDR. Thus, we next analyzed phosphorylation/activation of p53, as well as induction of p21^CIP1^, 24 and 48 h after exposure to 2.5 µM oxaliplatin. As shown in Figure 5A, the initial induction of p53 and its phosphorylation is comparable between SW48, SW480, and LoVo cells. Moreover, in HCT116^p53+/+^ cells, induction and phosphorylation was observed, which was however rather weak, as compared to the other cells. As expected, HCT116^p53−/−^ cells showed no expression of p53. Concerning expression of p21^CIP1^, SW48, SW480, LoVo and HCT116^p53+/+^, but not HCT116^p53−/−^ cells showed a pronounced induction. The data indicate that the initial induction of p53 and p21^CIP1^ is comparable between all p53-proficient cell lines and, therefore, cannot explain the differences in senescence induction between these cells. Next, we analyzed the persistence of p53 activation and p21^CIP1^ induction 120 h after oxaliplatin exposure, showing only in LoVo cells a sustained activation of p53 and upregulation of p21^CIP1^ (Figure 5B). Similar results were also observed at mRNA level. Whereas in LoVo cells a 20-fold induction of *p21^CIP1^* was observed, SW48, SW480, and HCT116^p53+/+^ cells showed only a 2–3-fold induction, and in HCT116^p53−/−^ cells the amount of *p21^CIP1^* mRNA was reduced (Figure 5C). The data suggest that LoVo cells utilize a specific mechanism reinforcing the induction of p53 response.

### 3.6. Expression of p14^ARF^ and p16^INK4A^ upon Oxaliplatin Treatment

**Figure 6:**
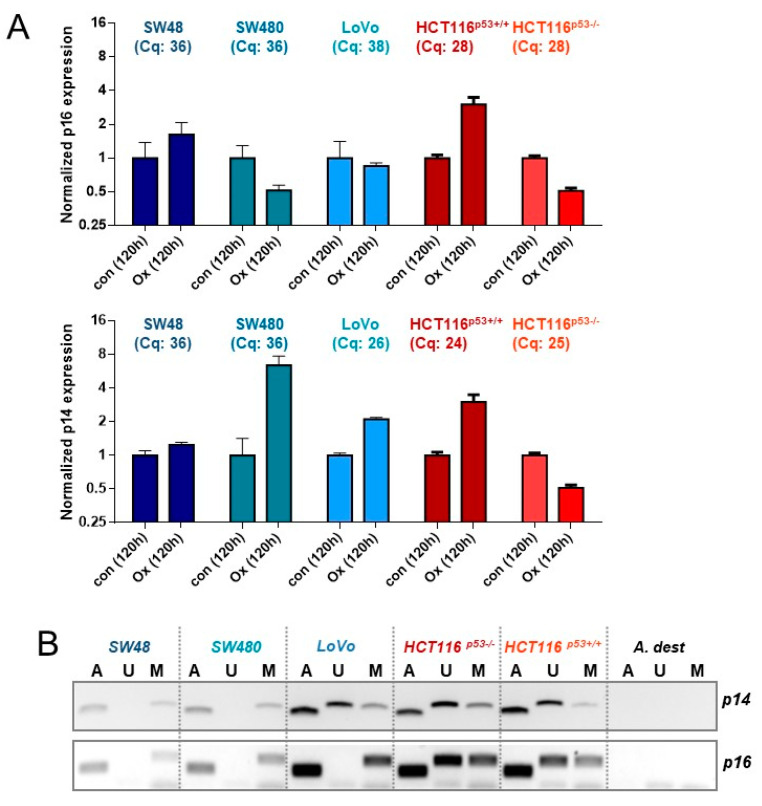

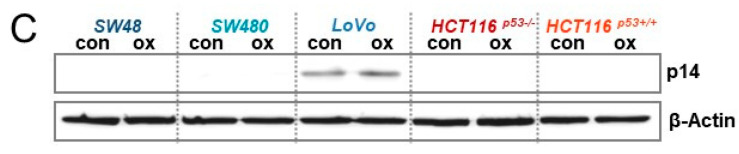
(**A**) CRC cells were treated with 2.5 µM oxaliplatin; 120 h later, the expression of *p14^ARF^* and *p16^INK4a^* mRNA was measured by qPCR. Cq values of the 120 h-control are provided for all cell lines. (**B**) Genomic DNA obtained from CRC cells was modified by bisulfite conversion and the 5meC methylation of *p14^ARF^ and p16^INK4a^* was measured by methylation-specific PCR (MSP). U=unmethylated, M = methylated, A = ACTB (positive control). (**C**) CRC cells were treated with 2.5 µM oxaliplatin. 120 h later, the expression of p14^ARF^ was determined by western-blot analysis. β-Actin was used as internal loading control.

Two crucial factors involved in senescence induction and its maintenance are p14^ARF^ and p16^INK4a^, which are encoded by different reading frames of the *CDKN2A* gene. Thus, we next analyzed the expression and induction of *p16^INK4a^* and *p14^ARF^* 120 h after exposure to oxaliplatin (Figure 6A). qPCR experiments showed a 3-fold upregulation of *p16^INK4a^* in HCT116^p53+/+^ cells but no induction in the other cell lines. In addition, striking differences were obtained at the basal level expression. Thus, comparing the Cq values revealed only in HCT116 cells a reliable expression of the *p16^INK4a^* mRNA (Cq: 28), whereas the Cq values for SW48, SW480, and LoVo (Cq: 36,36,38, respectively) were much higher, indicating a low *p16^INK4a^* expression. Similar results were obtained concerning *p14^ARF^* expression. In this case a strong *p14^ARF^* expression was observed in LoVo (Cq: 26) and HCT116 (Cq: 24, 25) cells. Apart from mRNA expression, we also analyzed the epigenetic silencing of *p14^ARF^* and *p16^INK4a^* (Figure 6B). The results obtained by methylation-specific PCR (MSP) revealed a complete methylation and, thereby, silencing of *p16^INK4a^* in SW48, SW480, and LoVo cells, whereas HCT116 cells were hemi-methylated. Since only SW48, SW480, and LoVo cells underwent senescence, an impact of p16^INK4a^ on senescence induction can be excluded. Of note, also HCT116 cells are p16^INK4a^ deficient, independent of mRNA expression; in HCT116 cells one allele is silenced via methylation, whereas the other allele is mutated, showing a base deletion in the codon 33 within the promoter/exon 1β region, generating a stop codon at codon 47 [33]. The same is also true for *p14^ARF^*in HCT116 cells. Interestingly, opposite to SW48 and SW480 cells, the *p14^ARF^* promoter of LoVo cells is hemi-methylated (Figure 6B). We should note that for SW48 and LoVo cells the *p14^ARF^* methylation and mRNA expression status is in line with the literature, whereas SW480 have been reported to be unmethylated [34], although in our hands, they are methylated for *p14^ARF^*. Since no mutations are known for *p14^ARF^* in LoVo cells, we verified the p14^ARF^ and p16^INK4a^ status by immunodetection, showing that among all CRC cell lines tested only LoVo express p14^ARF^ protein, indicating LoVo cells to be p14^ARF^ proficient (Figure 6C). Expression of p16^INK4a^ was observed in none of the cell lines (data not shown).

### 3.7. Impact of p53 and p14^ARF^ on Oxaliplatin-Induced Cellular Senescence

**Figure 7:**
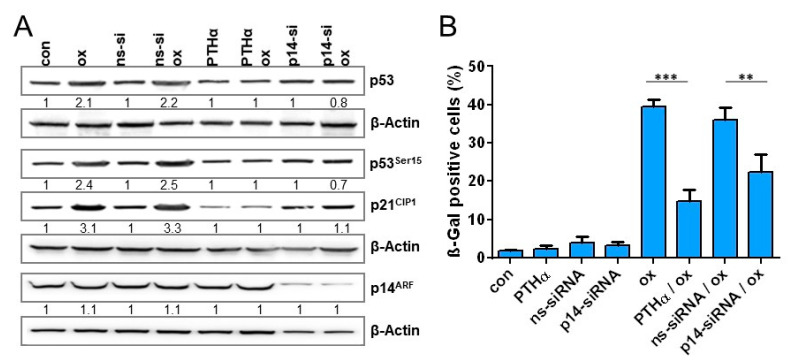

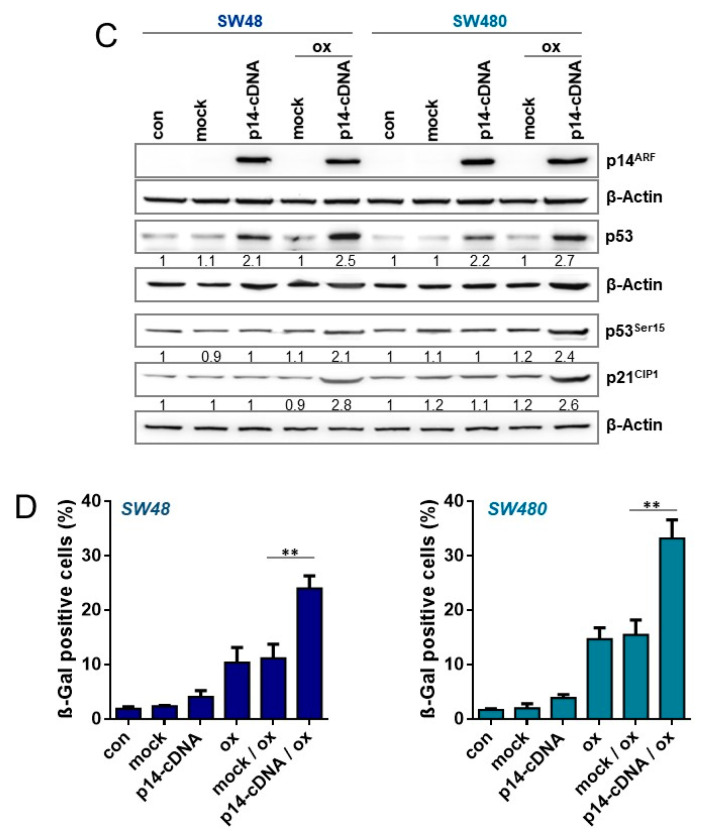
(**A**,**B**) LoVo cells were either exposed to PTHα (30 µM) or transfected with non-specific siRNA (ns-siRNA) or p14^ARF^ specific siRNA. Cells were treated with 2.5 µM oxaliplatin 8 h after siRNA or 1 h after PTHα treatment. (**A**) 120 h upon oxaliplatin exposure, the expression of p14^ARF^, p21^CIP1^, and p53, as well as the phosphorylation of p53 at Ser15 was measured by immunodetection. (**B**) Moreover, 168 h after oxaliplatin exposure, senescence was measured microscopically by detection of β-Gal positive cells. (**C**,**D**) SW48 and SW480 cells were transfected with a p14^ARF^ expression plasmid. Cells were non-treated or treated with 2.5 µM oxaliplatin 8 h after transfection. (**C**) 120 h upon oxaliplatin exposure, the expression of p14^ARF^, p21^CIP1^, and p53, as well as the phosphorylation of p53 at Ser15 was measured by immunodetection. (**D**) Furthermore, 168 h after oxaliplatin exposure, senescence was measured microscopically by detection of β-Gal positive cells. (**A**,**C**) β-Actin was used as internal loading control; x-fold induction was measured densitometrically and is annotated under the respective blot. (**B**,**D**) Experiments were performed in triplicates and differences between treatment and control were statistically analyzed using Student’s t test (not labeled = not significant, ** *p* < 0.01, *** *p* < 0.001).

To analyze whether the expression of p14^ARF^ is associated with sustained activation of the p53/p21^CIP1^ response in LoVo cells, p14^ARF^ was downregulated via siRNA. Furthermore, to verify that oxaliplatin-induced senescence requires p53, p53 was inhibited using 30 µM pifithrin α (PTHα). Under these conditions, inhibition of p53 completely blocked the oxaliplatin-induced increase in p21 expression, and knockdown of p14^ARF^ was accompanied by an abrogated oxaliplatin-induced stabilization and phosphorylation of p53 as well as by abrogated p21^CIP1^ induction (Figure 7A), indicating that p14^ARF^ is involved in stabilization and phosphorylation of p53 and, thereby, in the sustained upregulation of p21^CIP1^. Next, we measured the oxaliplatin-triggered induction of senescence upon inhibition of p53 and knockdown of p14^ARF^ (Figure 7B). As expected, inhibition of p53 nearly completely abolished senescence induction. Most importantly, also knockdown of p14^ARF^ significantly reduced onset of senescence to a level observed in SW48 and SW480 cells.

To analyze whether expression of p14^ARF^ could enhance senescence frequency in SW48 and SW480 cells, the cells were transiently transfected with a p14^ARF^ expression plasmid [14] (Figure 7C). Under these conditions, p14^ARF^ was detectable in SW48 and SW480 cells 120 h after transfection. The expression of p14^ARF^ was accompanied by an increased stabilization of p53 independent of oxaliplatin treatment. Opposite, enhanced phosphorylation of p53 as well as enhanced p21 induction was only observed in p14^ARF^ expressing SW48 and SW480 cells after oxaliplatin treatment. Similar to the phosphorylation of p53 and induction of p21^CIP1^, a significantly enhanced frequency of senescence was only observed in p14^ARF^ expressing SW48 and SW480 cells after oxaliplatin treatment (Figure 7D). Obviously, re-expression (rescue) of p14^ARF^ enhances stability of p53, however it seems not to be sufficient to induce senescence in absence of DNA damage. 

## 4. Discussion

Colorectal cancer (CRC) is caused by old age [13] and lifestyle factors, such as diet (red meat, processed meat, and alcohol) [19], smoking [23], obesity, and lack of physical activity, and is also associated with diseases, e.g., inflammatory bowel disease [14,15]. Both sexes combined, colon cancer comprises for 6.1% of all cancer cases and for 9.2% of cancer related deaths. In addition, rectal cancer comprises for 3.9% of all cancer cases and 3.2% of cancer related deaths [35,36]. Thus, CRC represents the third most frequent cancer and the second frequent cause of cancer-related death worldwide; the five-year survival rate amounts to 65% [35,36]. First-line therapy consists of fluoropyrimidines, such as 5-fluorouracil (5-FU) or capecitabine alone or in combination with leucovorin. Furthermore, either oxaliplatin (FOLFOX) or irinotecan (FOLFIRI) are co-administered [37,38]. The second-line therapy is performed by switching FOLFOX into FOLFIRI or the other way around [39]. FOLFOX-based therapy [40] has improved the response rate from 20 to 50% compared with 5-FU alone, and also the survival rate of metastatic patients [41,42]. During FOLFOX-based therapy, 85 mg oxaliplatin pro m^2^/body surface area is applied intravenous every two weeks for a total of 12 cycles.

Opposite to cisplatin, which is known to induce senescence in different tumor entities [43,44,45,46], the data concerning oxaliplatin-induced senescence are very limited. Thus, an induction of senescence was observed in the mouse cell line CT26 and in the rat cell line PROb [47]. Moreover, oxaliplatin treatment of tumor-bearing rats induced the expression of the SASP factors *IL6*, *IL8*, *CXCL1*, *CXCL2*, and *MMP* [47]. In human cancer cell lines, mostly negative results were obtained. Thus, HCT116 and SW480 cells induced senescence upon doxorubicin, 5-FU and irinotecan but not upon oxaliplatin exposure [48] and senescence could be strongly induced in various cancer cell lines (A549, SH-SY-5Y, HCT116, MDA-MB-231, and MCF-7) by doxorubicin, irinotecan, and methotrexate, whereas oxaliplatin and 5-FU failed to do so [49]. Therefore, it is not yet clear whether oxaliplatin can induce senescence in CRC cells and whether p53 is implicated in this pathway. Here, we show that oxaliplatin can induce senescence in CRC cells; however, this is highly cell line-specific.

To shed light on the question concerning oxaliplatin-induced senescence, we analyzed cell death, senescence induction as well as transcriptional activation of SASP factors and factors associated with SCAP and apoptosis in p53-proficient (p53 wild-type) SW48, HCT116^p53+/+^, and LoVo cells. Moreover, we included in the study SW480 cells, which harbor a p53 stabilizing mutation but retain p53-dependent transcriptional activity, and p53-deficient HCT116^p53−/−^ cells. All cell lines reacted to oxaliplatin with the induction of cell death, LoVo and HCT116^p53−/−^ cells exhibited the weakest response, whereas toxicity was most profound in HCT116^p53+/+^ cells. The difference between the isogenic HCT116 cells is in line with data showing that p53 sensitizes cells against oxaliplatin-induced cell death [50]. Irrespective of the cell line, the cells accumulated in the G2-phase of the cell cycle. Again, the weakest response was observed in LoVo cells. Correspondingly, the proliferation capacity determined via colony formation was most strongly abrogated in LoVo cells. In line with this, only LoVo cells showed induction of senescence, determined via β-Gal and C_12_FDG staining 120 h after oxaliplatin exposure. Similar results were obtained at later time points (168 h), at which the fraction of senescent cells was further enhanced in LoVo cells. At those later times, SW48 and SW480 cells also displayed a weak, but significant, induction of senescence. The induction of senescence observed in LoVo cells was accompanied by transcriptional activation of SASP components *CCL8*, *IL6*, and *IL8*, and of the anti-apoptotic SCAP factors *BIRC2* and *BIRC3*. Interestingly, in LoVo cells, the highest increase of different p53-dependent pro-apoptotic factors (*BAX*, *FASR*, *PUMA*, *NOXA*) and of the p53 target *MDM2* was observed, pointing to a more pronounced activation of the p53 pathway, in comparison with the other cell lines. 

To elucidate the molecular mechanism underlying the differential activation of senescence between the p53-proficient CRC cells, we compared activation of the DDR 24 and 48 h upon oxaliplatin. In all proficient cell lines, but not in HCT116^p53−/−^ cells, activation/phosphorylation of p53 and induction of p21^CIP1^ was observed at early time points after oxaliplatin treatment. Importantly, at later times (120 h), the activation/phosphorylation of p53 and induction of p21^CIP1^ was only observed in LoVo cells, indicating differential regulation of the p53/p21^CIP1^ pathway among the p53-proficient lines. 

**Figure A4:**
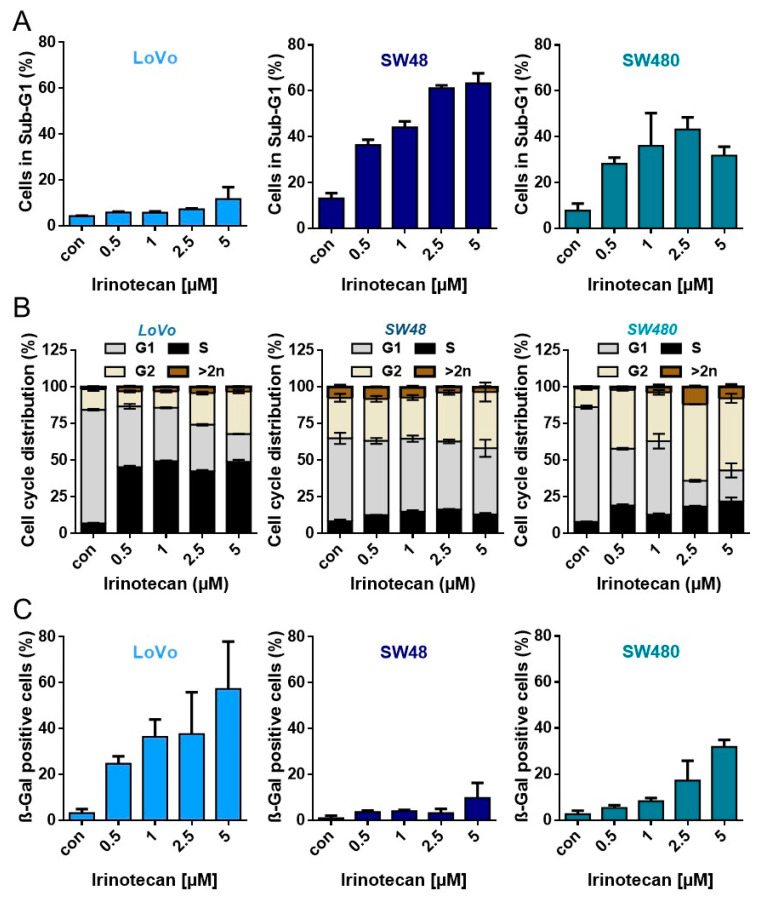
(**A**) Cell death was measured 168 h upon exposure to different concentrations of irinotecan by flow cytometry using PI staining in CRC cells. (**B**) Cell cycle distribution was measured 168 h upon exposure to different concentrations of irinotecan by flow cytometry using PI staining in CRC cells. (**C**) Senescence was measured microscopically by detection of β-Gal positive cells 168 h after irinotecan exposure.

Beside p21^CIP1^, p16^INK4a^ and p14^ARF^ also play important roles in cellular senescence. However, in oxaliplatin-induced senescence in CRC cells, an involvement of p16^INK4a^ could be excluded since all cell lines are p16^INK4a^ deficient. In contrast, we observed that LoVo cells are proficient for p14^ARF^, showing a hemimethylated promoter, as well as a strong expression of the mRNA and the protein. Opposite to p16^INK4a^, the role of p14^ARF^ in senescence is less clear. Thus, ectopic expression of p14^ARF^ induced a senescent phenotype in normal human fibroblasts [51] and p19^ARF^ (homolog of human p14^ARF^) deficient mouse fibroblasts do not show oncogenic H-Ras induced senescence [52]. However, the impact of p14^ARF^ may differ between human and rodent fibroblasts, at least during oncogenic senescence. Hence, p14^ARF^ was upregulated by c-Myc in senescent MEFs [53], but not upon H-Ras in human fibroblasts [54]. Moreover, ectopic expression of p14^ARF^ was shown to arrest p21^CIP1^ deficient MEFs [55], but not p21^CIP1^ deficient human fibroblasts [54]; Ras can upregulate p14^ARF^ in rodent cells [56,57], but not in human fibroblasts [54]. Since it was previously shown that c-Myc-induced senescence in human primary cells requires stabilization of p14^ARF^ [58], the observed differences can also be caused by differences in the oncogenes used for senescence induction. Concerning the impact of p14^ARF^ on DNA damage-induced senescence it has been suggested that p14^ARF^ plays a role in the long-term maintenance of p53 activity following ionizing radiation [59]; however, its expression seems not to be dependent on DNA damage [15]. Our data suggest that p14^ARF^ may be important for oxaliplatin-induced senescence in CRC cells. Therefore, the impact of p14^ARF^ on p53 stability and senescence maintenance was analyzed by knockdown approach. As positive control, p53 was inhibited. The data clearly show that p53 inhibition nearly completely blocks oxaliplatin-dependent p21^CIP1^ expression and senescence. Importantly, knockdown of p14^ARF^ abrogated oxaliplatin dependent expression/activation of p53 and of p21^CIP1^ at later times and significantly reduced senescence to a level similar to SW48 and SW480. *Vice versa*, ectopic expression of p14^ARF^ enhanced oxaliplatin-induced senescence in SW48 and SW480 cells. Of note, the importance of p14^ARF^ for anticancer drug-induced senescence seems not to be restricted to oxaliplatin since, in the case of the topoisomerase I (TOP1) inhibitor irinotecan LoVo cells showed the strongest induction of senescence (Figure A4).

Overall, our data clearly show that the assumption that oxaliplatin cannot induce senescence in CRC cells is not true. We observed induction of senescence in three out of four p53-proficient CRC lines. However, the efficiency in inducing senescence strongly differed between these cell lines. Thus, we could show that proficiency in p14^ARF^ mediates sustained p53 activity/expression and p21^CIP1^ expression and, thereby, reinforces senescence induction. Of note, in parallel also an increased activation of p53-dependent pro-apoptotic pathways was observed. However, the p53-dependent activation of apoptosis seems to be efficiently blocked in LoVo cells, which could be explained by the strong induction of *BIRC2*/c-IAP1 and *BIRC3*/c-IAP2, probably representing the central component of the SCAP during oxaliplatin-induced senescence. This, however, has to be addressed in future work. Our data also suggest that p14^ARF^ might influence the decision between cell death and senescence in primary CRC. Moreover, differential epigenetic silencing of p14 ^ARF^ and/or p16 ^INK4A^ among cell lines from different labs could explain the heterogeneity in the data published concerning anticancer-drug-induced senescence. Opposite to cell lines, most primary CRCs are p14^ARF^ proficient. Thus, meta-analysis showed p14^ARF^ methylation in ~25% of all cases [60]. The impact of p14^ARF^ on chemotherapy-induced senescence in CRC will be an important topic for future studies.

## Data Availability

Data is contained within the article.

## Appendix A

**Table A1:** DNA primers.

Gene	Primer Sequence (5‘-3‘)
ACTB-fw	TGGCATCCACGAAACTACC
ACTB-rev	GTGTTGGCGTACAGGTCTT
BAX-fw	CAGAAGGCACTAATCAAG
BAX-rev	ATCAGATGTGGTCTATAATG
Bcl2-fw	TTCAGAGACAGCCAGGAGAAA
Bcl2-rev	AGTACCTGAACCGGCACCT
BCLx_L_-fw	AAGCGTAGACAAGGAGAT
BCLx_L_-rev	TAGGTGGTCATTCAGGTAA
BIRC2/c-IAP1-fw	TTCCCAGGTCCCTCGTATCA
BIRC2/c-IAP1-rev	CCGGCGGGGAAAGTTGAATA
BIRC3/c-IAP2-fw	TCACTCCCAGACTCTTTCCA
BIRC3/c-IAP2-rev	CCCCGTGTTCTACAAGTGTC
BIRC5/Survivin-fw	ATGACTTGTGTGTGATGA
BIRC5/Survivin-rev	GTTTGTGCTATTCTGTGAA
CCL2-fw	CCTTCATTCCCCAAGGGCTC
CCL2-rev	CTTCTTTGGGACACTTGCTGC
CCL8-fw	TGTCCCAAGGAAGCTGTGAT
CCL8-rev	TGGAATCCCTGACCCATCTCT
CXCL1-fw	CTGGCTTAGAACAAAGGGGCT
CXCL1-rev	TAAAGGTAGCCCTTGTTTCCCC
IL-1α-fw	TCTTCTGGGAAACTCACGGC
IL-1α-rev	GCACACCCAGTAGTCTTGCT
IL-1β-fw	TGAGCTCGCCAGTGAAATGA
IL-1β-rev	AGATTCGTAGCTGGATGCCG
IL-6-fw	GCTGCAGGACATGACAACTC
IL-6-rev	AACAACAATCTGAGGTGCCC
IL-8-fw	CCAAACCTTTCCACCCCAAA
IL-8-rev	CTCTGCACCCAGTTTTCCTT
FASR-fw	AGAACTTGGAAGGCCTGCAT
FASR-rev	CTGGTTCATCCCCATTGACT
FASL-fw	GGGATGTTTCAGCTCTTCCA
FASL-rev	TAAATGGGCCACTTTCCTCA
MDM2-fw	ATCTTGATGCTGGTGTAA
MDM2-rev	AGGCTATAATCTTCTGAGTC
PUMA-fw	GACGACCTCAACGCACAGTA
PUMA-rev	CTGGGTAAGGGCAGGAGTC
NOXA-fw	ACACGGTGGACGTCCTGT
NOXA-rev	ACGAAGCACTTGGGGAAGAT
p14-fw	CCCTCGTGCTGATGCTACTG
p14-rev	CATCATGACCTGGTCTTCTAGGAA
p16-fw	GGAGCAGCATGGAGCCTTC
p16-rev	CATCATCATGACCTGGATCG
p21-fw	TACATCTTCTGCCTTAGT
p21-rev	TCTTAGGAACCTCTCATT
TNFα-fw	CAGCCTCTTCTCCTTCCTGAT
TNFα-rev	GCCAGGGGCTGATTAGAGA
XIAP-fw	CCGAAGAGAAACCACATTT
XIAP-rev	CTGAGCCAGATCAAAGTATG

**Table A2:** Antibodies.

Protein	Molecular Weight (kDa)	Antibody	RRID	Company
ß-Actin	42	sc-47778	AB_2714189	Santa Cruz Biotechnology
HSP90	90	sc-13119	AB_675659	Santa Cruz Biotechnology
p14	14	sc-53640	AB_785015	Santa Cruz Biotechnology
p21	21	sc-817	AB_628072	Santa Cruz Biotechnology
p53	53	sc-100	AB_628087	Santa Cruz Biotechnology
p53^Ser15^	53	#9286	AB_331741	Cell Signaling Technology
HRP conjugated anti-mouse		KCB002	AB_10703407	Rockland
HRP conjugated anti-rabbit		KCB003	AB_10702763	Rockland
